# Superconducting spin reorientation in spin-triplet multiple superconducting phases of UTe_2_

**DOI:** 10.1126/sciadv.adg2736

**Published:** 2023-07-28

**Authors:** Katsuki Kinjo, Hiroki Fujibayashi, Hiroki Matsumura, Fumiya Hori, Shunsaku Kitagawa, Kenji Ishida, Yo Tokunaga, Hironori Sakai, Shinsaku Kambe, Ai Nakamura, Yusei Shimizu, Yoshiya Homma, Dexin Li, Fuminori Honda, Dai Aoki

**Affiliations:** ^1^ Department of Physics, Graduate School of Science, Kyoto University, Kyoto 606-8502, Japan.; ^2^ Advanced Science Research Center, Japan Atomic Energy Agency, Tokai, Ibaraki 319-1195, Japan.; ^3^ Institute for Materials Research, Tohoku University, Oarai, Ibaraki 311-1313, Japan.; ^4^ Central Institute of Radioisotope Science and Safety, Kyushu University, Fukuoka 819-0395, Japan.; ^5^ University Grenoble Alpes, CEA, Grenoble INP, IRIG, PHELIQS, F-38000 Grenoble, France.

## Abstract

Superconducting (SC) state has spin and orbital degrees of freedom, and spin-triplet superconductivity shows multiple SC phases because of the presence of these degrees of freedom. However, the observation of spin-direction rotation occurring inside the SC state (SC spin rotation) has hardly been reported. Uranium ditelluride, a recently found topological superconductor, exhibits various SC phases under pressure: SC state at ambient pressure (SC1), high-temperature SC state above 0.5 gigapascal (SC2), and low-temperature SC state above 0.5 gigapascal (SC3). We performed nuclear magnetic resonance (NMR) and ac susceptibility measurements on a single-crystal uranium ditelluride. The *b* axis spin susceptibility remains unchanged in SC2, unlike in SC1, and decreases below the SC2-SC3 transition with spin modulation. These unique properties in SC3 arise from the coexistence of two SC order parameters. Our NMR results confirm spin-triplet superconductivity with SC spin parallel to *b* axis in SC2 and unveil the remaining of spin degrees of freedom in SC uranium ditelluride.

## Teaser

Multiple superconducting phases with spin rotation in the superconducting state was observed.

## INTRODUCTION

What kind of an ordered state is realized under competing interaction is a central issue in condensed matter physics. Superconductivity and superfluidity are most remarkable macroscopic quantum phenomena produced by two fermion pairs, called the Cooper pair. In conventional superconductivity, which covers almost all superconductors found so far, the Cooper pairs have zero total spin and orbital angular momenta and have no degrees of freedom. Therefore, only one superconducting (SC) state is realized.

Theoretically, it is possible that either or both of the two angular momenta are nonzero, and hence, the coexistence of two SC phases, and/or multiple SC phases, was anticipated. The well-known example of such multiphases is the superfluid ^3^He, in which two phases (A and B) with the lowest energies are realized in zero field and becomes more complex under magnetic fields and/or anisotropic environment (*1*–*4*). The presence of the Majorana particles, which is applicable to “qubits” in quantum computer, was suggested in the surface state of the B phase (*5*). However, in superconductors, there are only limited examples for such multiple SC phases, for example, UPt_3_ (*6*), Th-doped UBe_13_ (*7*), and CeRh_2_As_2_ (*8*) are known so far. In addition, SC spin rotation, which is the smoking gun for the spin-triplet SC multiphase due to the presence of the spin degrees of freedom, has hardly been observed because of the tiny difference between the two critical temperatures (*9*). It is quite important to find textbook examples of the spin-triplet superconductivity, corresponding to the superfluid ^3^He, and to find the phenomena related to the spin and orbital degrees of freedom.

**Fig. 1.**
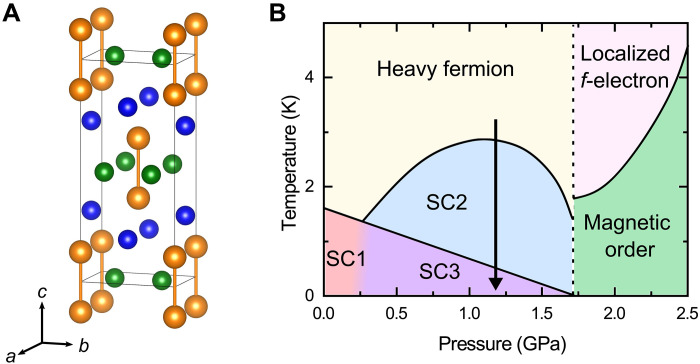
Crystal structure and pressure-temperature phase diagram of UTe_2_. (**A**) Crystal structure of UTe_2_ made by VESTA (*39*). The orange ball represents the U atom. The blue and green balls represent the Te(1) and Te(2) atom respectively. The U atom forms the dumbbell structure, and the dumbbells form a line along the *a* axis. (**B**) Pressure-temperature phase diagram of UTe_2_. Bold lines correspond to the thermodynamic phase transitions detected by the specific heat measurements (*11*, *13*). The phase boundary between SC1 and SC3 has not been detected. The arrow represents the measurement range in this report.

Here, we focus on a recently found uranium-based superconductor uranium ditelluride (UTe_2_) with SC transition temperature *T*_c_ ∼ 1.6 K (*10*). UTe_2_, crystallizing orthorhombic structure with space group *Immm* (#71, *D*_2*h*_), as shown in Fig. 1A, has multiple SC phases. Under pressure (*P*), the *T*_c_ of UTe_2_ increases to about 3 K at 1.2 GPa (*11*–*13*), as shown in Fig. 1B. Below 1.6 GPa, the two jumps in the temperature (*T*) dependence of specific heat indicate the existence of at least two SC phases (*11*, *13*): One exists at ambient pressure (SC1) and its *T*_c_ gradually decreases with applying *P*, and the other is an SC phase induced by *P* above 0.5 GPa (SC2), whose *T*_c_ has a maximum at 1.2 GPa. As a result, *T*_c_ of SC1 is below *T*_c_ of SC2 above 0.5 GPa, and thus, we call this SC state SC3 in this paper. Above 1.6 GPa, superconductivity suddenly disappears and a magnetic anomaly, which is considered as the antiferromagnetic state, was observed (*13*).

In addition, UTe_2_ shows multiple SC phases even at ambient pressure. For *H*||*b*, *T*_c_ first decreases with increasing *H* and shows a minimum at around 16 T and then *T*_c_ increases up to 35 T (*14*). The bulk SC properties in the high-field SC (HFSC) phase were recently confirmed by thermodynamic and χ_ac_ measurements (*15*, *16*), suggesting that the pairing interaction grows up above 16 T (*15*).

Furthermore, recent nuclear magnetic resonance (NMR) measurements revealed a tiny change of spin susceptibility just below *T*_c_ for *H*||*b* and *H*||*c* (*17*–*19*), and almost no change in the *a* axis spin susceptibility (*20*). Above 14 T for *H*||*b*, spin susceptibility remains constant below *T*_c_ (*16*, *18*). These results indicate that UTe_2_ is a spin-triplet superconductor with spin degrees of freedom and SC order parameter **d** vector, which is perpendicular to the SC spin, having the *b* and *c* components in the low-field (LF) region. In UTe_2_, a complex interplay of various interactions was reported, such as Ising-like ferromagnetic fluctuations from NMR studies (*21*) and the antiferromagnetic fluctuations from the inelastic neutron scattering measurements (*22*, *23*). Such competing interactions are thought to induce rich physics such as the HFSC phase in *H*||*b* and multiple SC phases under pressure. Thus, identifying the SC properties of the SC2 and SC3 states is the last piece to understanding the multiple SC phases of UTe_2_.

To elucidate the SC properties of the multiple SC phases in UTe_2_, NMR serves as a highly effective tool. NMR is a technique that probes nuclear spins, which interact with electron spins through strong hyperfine interactions. Consequently, it enables us to investigate the electron spin susceptibility microscopically. While the spin susceptibility in the SC state cannot be measured because of SC diamagnetism by the bulk magnetization measurement, NMR can detect it with the hyperfine interaction. Moreover, to measure spin susceptibility in multiple SC states under pressure, NMR is a unique measurement that meets these requirements.

Here, we report the results of the NMR Knight shift *K* for *H*||*b* (*K_b_*) at 1.2 GPa, at which *T*_c_ of SC2 is maximum. We found that the spin susceptibility along the *b* axis is unchanged in SC2, which is different from that observed in SC1 at ambient pressure. More unexpectedly, at the SC2-SC3 transition, *K_b_* suddenly decreases and the NMR spectral width becomes broader. These results suggest that the SC order parameter of SC1 and SC2 is different and that SC2 is a spin-triplet superconductivity with spin oriented toward the *b* axis and SC spin reoriented in SC3 state. These results suggest that the SC order parameter **d** vector was changed at the SC3 transition, and the spin degrees of freedom remain in the superconductivity of UTe_2_.

## RESULTS AND DISCUSSION

**Fig. 2.**
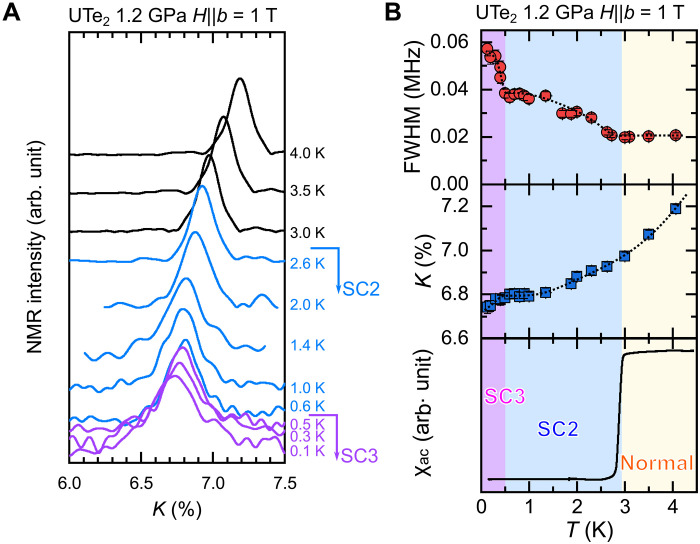
NMR evidence for SC spin rotation. (**A**) Temperature variation of the NMR spectra of UTe_2_ at 1.2 GPa. (**B**) Temperature dependence of the FWHM, peak position *K* of Te(2) signal, and χ_ac_ at 1.2 GPa. Line colors in (A) and background color in (B) represent the phase appearing in Fig. 1B. The kinks in the *T* dependence of *K* and FWHM suggest the phase transition at around 0.5 K, although χ_ac_ does not show any anomaly. The dashed lines at the top and middle are guides to eye.

**Fig. 3.**
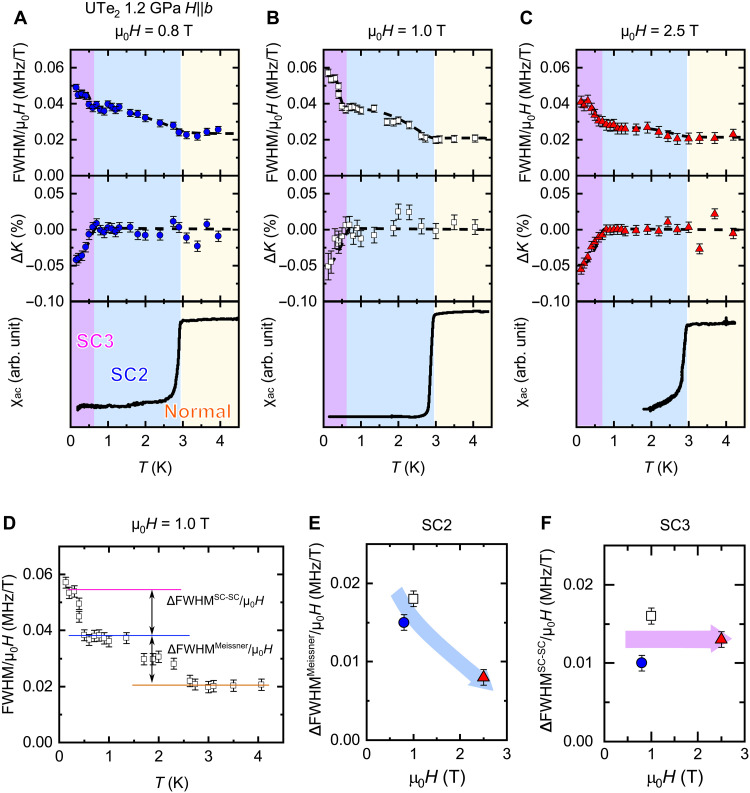
NMR data evidencing the increase in magnetic inhomogeneity. Temperature dependence of (top) the FWHM/μ_0_*H*, (middle) Δ*K*, and (bottom) χ_ac_ at (**A**) 0.8 T, (**B**) 1.0 T, and (**C**) 2.5 T of the Te(2) NMR spectrum. (**D**) The definition of ΔFWHM^Meissner^/μ_0_*H* and ΔFWHM^Meissner^/μ_0_*H*, which are the NMR linewidth broadening in SC2 and SC3, respectively, is shown. The magnetic field dependence of (**E**) ΔFWHM^Meissner^/μ_0_*H* and (**F**) ΔFWHM^SC-SC^/μ_0_*H*. The definitions of these two are given in the main text. ΔFWHM^Meissner^/μ_0_*H* decreases with applying field. This is conventional SC behavior and evidences the bulk superconductivity in the SC2 state. ΔFWHM^SC-SC^/μ_0_*H* is almost constant against the field.

As there are two crystallographically inequivalent Te sites in UTe_2_, we observed two ^125^Te-NMR peaks as reported in the previous paper (*17*–*19*): an NMR peak with the smaller (larger) *K* in *H*||*b* as Te(1) [Te(2)] by following the previous paper (*21*). The *T* variation of the NMR spectrum of Te(2) at *P* = 1.2 GPa is shown in Fig. 2A, and the *T* dependence of the full width at half maximum (FWHM), the NMR Knight shift (*K*) determined with the NMR spectrum, and χ_ac_ are shown in Fig. 2B. Here, the NMR Knight shift is proportional to the spin susceptibility, and the FWHM is proportional to the distribution of magnetization. As reported in the previous papers (*24*–*26*), *K_b_* exhibits a broad maximum at *T*χ_max_ and gradually decreases with decreasing *T*, similar to the *T* dependence of χ (*24*). Owing to the increase in *T*_c_ and the decrease in *T*χ_max_ with increasing *P* (*24*), *K_b_* highly depends on *T* around *T*_c_ at 1.2 GPa, unlike the case of ambient pressure (0 GPa). To clarify the change in the Knight shift related to the SC transition, we calculate ∆*K* ≡ *K*_SC_^spin^ − *K*_normal_^spin^ as shown in Fig. 3A. Here, *K*_normal_^spin^ (*K*_SC_^spin^) is *K*^spin^ in the normal (SC) state and is estimated from the extrapolation above *T*_c_. Although the absolute value of the spin part in *K* (*K*_spin_) decreases with decreasing *T*, ∆*K* is almost zero above 0.5 K, indicating that the spin susceptibility in SC2 is the same as that in the normal state for *H*||*b*. This *T* dependence of the spin susceptibility in SC2 is similar to that in the A phase of the ^3^He superfluid (*27*). Below 0.5 K, we observed a sudden decrease in *K_b_* and a broadening of the NMR spectrum, although no additional change was observed in χ_ac_. The magnitude of the decrease in *K_b_* below 0.5 K is almost the same as that in *K* at 0 GPa (*17*). This is further evidence of the occurrence of the phase transition inside the SC state from the microscopic point of view.

Now, we consider possible SC order parameters suggested by the present NMR results. As discussed in the previous papers (*17*–*20*), the order parameter of spin-triplet superconductivity is **d** vector, which is perpendicular to the SC spin components. In other words, *K_i_* proportional to the spin susceptibility along the *i* axis decreases when **d** vector has the *i* component, but is unchanged when the **d** vector has no *i* component. This corresponds to the SC spin pointing to the *i* direction. The possible SC phases at zero field are listed in table 1 of (*16*). Our findings indicate that the **d** vector has no *b* component (the SC spin is oriented to the *b* axis) in SC2 and has a finite *b* component in SC3, which is the same as that of SC1. This is the **d** vector rotation, evidencing the spin-triplet multiple SC phases with spin degrees of freedom.

**Fig. 4.**
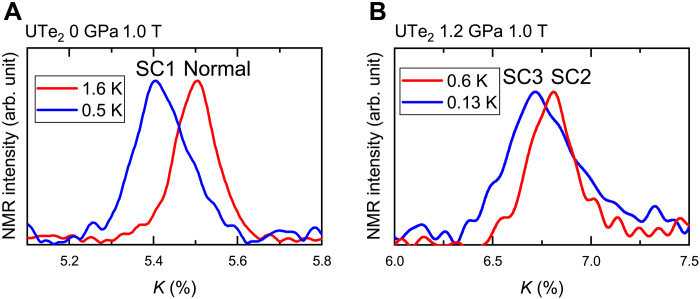
Comparison of the NMR spectrum broadening in SC1 and SC3. (**A**) The NMR spectrum variation occurring in SC1. (**B**) The NMR spectrum broadening occurring in SC3. The spectrum in SC3 shows a tail to the larger Knight shift side than the Knight shift in SC2.

Another important point of Fig. 2B is that the FWHM of the Te(2) site shows an additional increase below 0.5 K. Figure 3 (A to C) shows the temperature dependence of FWHM divided by the applied fields (μ_0_*H*), which reflects the distribution of the spin susceptibility, ∆*K* measured at the Te(2) site, and χ_ac_ measured at 0.8, 1.0, and 2.5 T, respectively. To clarify the increase in linewidth due to the normal-SC2 transition and the increase in linewidth due to the SC2-SC3 transition, we defined the values ΔFWHM^Meissner^ and ΔFWHM^SC-SC^. ΔFWHM^Meissner^ and ΔFWHM^SC-SC^ are defined as FWHM(SC2) − FWHM(Normal) and FWHM(SC3) − FWHM(SC2), respectively, as shown in Fig. 3D. In general, the FWHM of the NMR spectrum increases below *T*_c_ because of SC diamagnetism. This effect is related to the SC penetration depth, was observed below 3.0 K in SC2, and was suppressed with increasing μ_0_*H*, as shown in Fig. 3E. Decrease of ΔFWHM^Meissner^/μ_0_*H* with applying *H* is interpreted by the conventional SC diamagnetic effect. However, the additional increase in the FWHM below 0.5 K (ΔFWHM^SC-SC^/μ_0_*H*) cannot be explained by such SC diamagnetic effect, because χ_ac_ shows no anomaly around 0.5 K, and ΔFWHM^SC-SC^/μ_0_*H* is independent of the applied field, as shown in Fig. 3F. In addition, from the comparison of the NMR spectra between 0.6 K (SC2) and 0.13 K (SC3), it is noted that the spectrum shows a tail to the larger Knight shift side than the Knight shift in SC2 as shown in Fig. 4B. This is quite unusual for the NMR spectrum in the SC state, as the spin susceptibility decreases in the SC state. Such an NMR spectrum variation in the SC state was not observed in SC1 (Fig. 4A), although the spectrum broadening due to the SC diamagnetic effect was observed. The ratio between the FWHM of the Te(1) and Te(2) spectra in SC3 is almost unchanged to that in the normal state (*28*). These results indicate that the additional linewidth broadening in SC3 arises from the unusual inhomogeneity of the spin susceptibility, which was not observed in SC1. Thus, SC3 is a different SC state from SC1.

We consider the several possibilities of the origin of linewidth broadening in SC3. One is due to the SC vortex state in SC3. Because spin susceptibility is unchanged in SC2, the NMR signal from the vortex core is observed at almost the same position as the SC NMR signal outside the vortex. On the other hand, the spin susceptibility decreases in SC3, and the signal from the vortex core should be observed at the different position from the SC NMR signal, resulting in the NMR signal becoming broader in the mixed state of SC3. However, this possibility would be unlikely, because the measurement fields are far below *H*_c2_^orb^ ~ 70 T expected from the Werthamer, Helfand, and Hohenberg theory (*29*). Thus, the area fraction of the vortex is approximately 1%, and the contribution to the NMR spectrum is negligibly small. Another possibility is spatial inhomogeneity with the coexistence of the two SC states in the sample. However, this possibility is ruled out, as the observed spectra could not be explained by the simple coexistence of the SC2 and SC3 states (*28*). These results indicate that, whatever the inhomogeneities in the sample, the NMR linewidth broadening intrinsically occurs below the SC2-SC3 transition and is proportional to the magnetic field. In other words, it represents an increase in the distribution of the magnetic susceptibility in the SC3 state. At present, we have no clear interpretation of this phenomenon, but we believe that we observed an effect because of the mixing of the two SC order parameters as in the case of (U,Th)Be_13_ (*30*, *31*). Actually, it was pointed out that the nonunitary spin-triplet superconductivity leads to a spin-density wave-type modulation of **d** vector from theoretical studies (*32*).

**Fig. 5.**
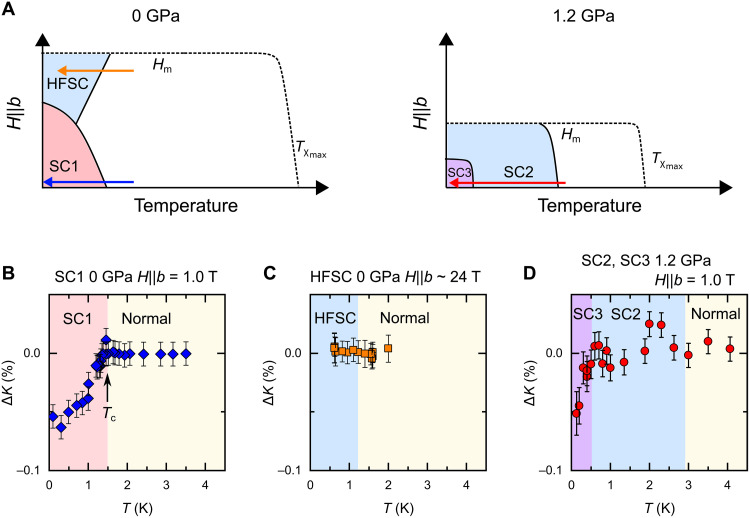
General phase diagram expected from our NMR results. (**A**) The schematic images of the *H-T* phase diagram of UTe_2_ at 0 and 1.2 GPa. The bold lines are the transition lines determined by the thermodynamic measurements (*11*, *13*, *15*). The dashed line represents the temperature where the χ*_b_* shows maximum (*T*_χmax_) and metamagnetic transition. (**B** to **D**) The temperature dependence of Δ*K* in four states appears in (A). The colored arrows in (A) represent the measurement range in (B) to (D).

Figure 5 (A and B) shows the schematic image of *T* versus *H* along the *b* axis (*H_b_*) phase diagram of UTe_2_ at ambient pressure (Fig. 5A) and 1.2 GPa (Fig. 5B) (*13*, *15*, *18*, *26*). At ambient pressure, *T*_c_(*H*) decreases in the LFSC (low-field SC) state and increases in the HFSC state up to the metamagnetic field 35 T (*H*_m_). In the LFSC state, *K_b_* slightly decreases below *T*_c_ (see Fig. 5B) (*17*). As mentioned above, *K_b_* is unchanged in the HFSC state (*16*), as shown in Fig. 5C, indicating that the SC spin aligns to the *b* axis. At the *P* ∼ 1.2 GPa where *T*_c_ becomes maximum, *H*_m_ is suppressed to almost half of *H*_m_ at *P* = 0, and there is no more L-shaped behavior in *H_b_*-*T* phase diagram (*26*). Considering the similarity in spin susceptibility in the SC state between the HFSC and SC2 states and first-order transition such as disappearance of SC state and Kondo coherent state with applying field or pressure (*25*, *33*, *34*), we conclude that the HFSC evolves into SC2 with applying pressure. In addition, the SC1 phase hides under the SC2 phase and becomes the SC3 phase (coexisting phase of SC1 and SC2), while the SC3 phase appears only inside the SC2 phase.

**Fig. 6.**
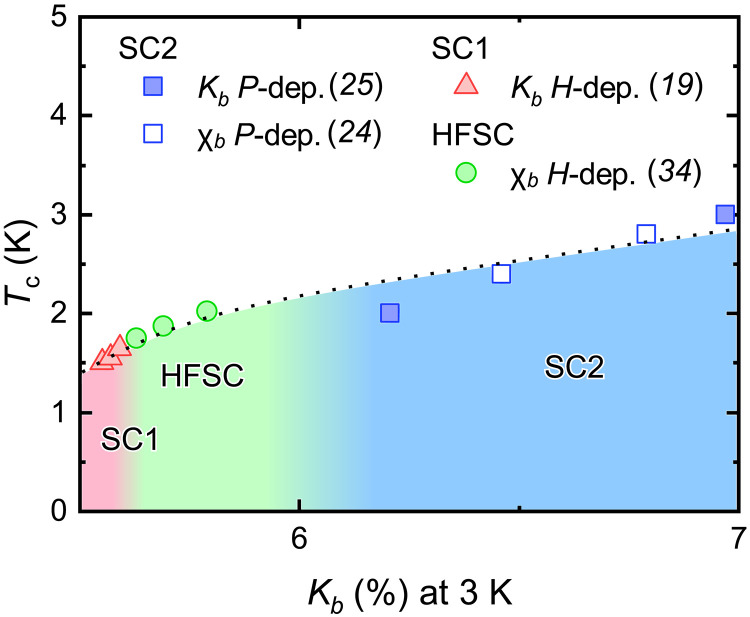
Relation between *b* axis susceptibility and critical temperature. This diagram shows the relation between *b* axis magnetic susceptibility and the *T*_c_ of SC phases. For *K_b_* between 5.7 and 6.0, *T*_c_ is affected by the magnetic field along the *b* axis. The extrapolation of *T*_c_ to *H* = 0 in HFSC is based on the strong coupling parameter of Miyake *et al.* (*34*). The magnetic field enhances *K_b_* and increases the *T*_c_. As *K_b_* increases further, *T*_c_ enters the SC2 region, and when *K_b_* increases to nearly 7%, *T*_c_ increases to a maximum of 3 K. The dotted line is a guide to eyes.

Another similarity between HFSC and SC2 is that *T*_c_ increases with increasing *K_b_*; Fig. 6 shows the values of *K_b_* under magnetic field or under pressure. When a magnetic field is applied, the zero-field extrapolated *T*_c_ continues to increase with the application of the field (*34*), and *K_b_* also increases. This tendency was also observed in the pressure experiment. When *K_b_* is above 6.0%, indicating the sample in SC2, *T*_c_ increases up to 3 K with increasing *K_b_*. The *T*_c_ versus *K_b_* curves show similarity between under pressure and under magnetic field. It strongly suggests that HFSC and SC2 are similar phases and have the same origin. On the basis of this scenario, we suggest that the spin susceptibility along the *b* axis (χ*_b_*) is an important parameter for HFSC and SC2, which determine the *T*_c_ of these states.

In conclusion, we have performed ^125^Te-NMR measurement on UTe_2_ under pressure to investigate the SC properties and determine the SC order parameter of SC2 and SC3 phases. The *b* axis spin susceptibility in SC2 has the same value as that of the normal state below 3.0 K above 0.5 K. Below 0.5 K, spin susceptibility decreases, and the NMR linewidth increases. These results indicate that the SC order parameter is changed, i.e., SC spin rotation occurs at the SC2-SC3 transition and the novel SC state with two SC order parameters in SC3. This is microscopic evidence for the remaining the spin degrees of freedom in the superconductivity of UTe_2_, inherent to the spin-triplet superconductivity.

## MATERIALS AND METHODS

### Sample preparation

A high-quality single-crystal UTe_2_ sample was grown by the chemical vapor transport method, with iodine as a transport agent, details of which are described in (*10*, *35*). The single crystal of UTe_2_ was an almost rectangular shape with 2 mm by 1 mm by 1 mm, with 2 mm along the *a* axis. To improve ^125^Te-NMR signal intensity, the sample was synthesized with the ^125^Te-enriched (99.9%) metal as discussed in (*18*). The *T*_c_ of this sample is 1.6 K at ambient pressure.

### ac susceptibility measurements

To confirm *T*_c_, we measured the high-frequency ac susceptibility χ_ac_ using the NMR tank circuit. In the SC state, the impedance of the circuit changes because of the Meissner effect, and thus, the tuning frequency of the circuit markedly changes just below *T*_c_.

### Applying pressure and pressure estimation

The hydrostatic pressure is applied using a piston cylinder–type cell made of NiCrAl and CuBe alloys as described in (*36*). Daphne 7373 was used as a pressure medium. The applied pressure was estimated by the SC transition temperature of Pb with the formula of *P* = [7.181 − *T*_c_(*P*)]/0.364 (*37*).

### Field alignment

We used a split-pair magnet to apply horizontal fields to the sample and a ^3^He─^4^He dilution refrigerator that can be rotated about the vertical axis. With this setup, we could rotate the magnetic field direction within the *bc* plane of the sample. The direction of the magnetic field was confirmed by measuring the Te(1) signal as described in (*24*).

### NMR measurements

An NMR spectrometer with a 100 W (at 0 dB input) power amplifier (Thamway, product: N146-5049A) was used for the measurements. A conventional spin-echo method was used for NMR measurements in the temperature range from 0.1 to 4.2 K and in the magnetic field range from 0.8 to 2.5 T. All measurements were carried out with the ^3^He─^4^He dilution refrigerator, in which the pressure cell was immersed into the ^3^He─^4^He mixture to reduce radio frequency heating during measurements. The NMR spectrum was obtained by summation of the fast Fourier transform spectrum with a 5 kHz step. The applied field is estimated by ^65^Cu-NMR measurements as discussed here (*38*).

## Supplementary Materials

### This PDF file includes:

Supplementary Text Figs. S1 to S4 References
