# Supplementary material for: Superconducting spin reorientation in spin-triplet multiple superconducting phases of UTe2
Source: Sci Adv. 2023 Jul 28;9(30):eadg2736. doi: 10.1126/sciadv.adg2736 (PMC10381943; doi:10.1126/sciadv.adg2736)
Supplement: Supplementary file 1 — Supplementary Text Figs. S1 to S4 References [file sciadv.adg2736_sm.pdf]

Science Advances
AAAS

Supplementary Materials for

Superconducting spin reorientation in spin-triplet multiple superconducting phases of UTe_2

Katsuki Kinjo *et al.*

Corresponding author: Katsuki Kinjo, katsuki.kinjo.c6@tohoku.ac.jp; Kenji Ishida, kishida@scphys.kyoto-u.ac.jp

Sci. Adv. **9**, eadg2736 (2023)
DOI: 10.1126/sciadv.adg2736

This PDF file includes:

Supplementary Text
Figs. S1 to S4
References

Supplementary Text

Heat-up effect by NMR Radio-Frequency pulses

The superconductivity just after the Radio-Frequency (RF) pulses for the NMR-signal observation was confirmed by the following heat-up test using the same set-up as the NMR measurements with the pressure cell at 1.2 GPa (*40-42*). We applied a weak RF pulse with a pulse width of 30 μs and the same frequency as the NMR measurements and measured the voltage of the real (Re) and imaginary (Im) part of the output signal voltage from the NMR receiver. We call this weak RF pulse "phase-detection pulse (PDP)".

First, we measured the T dependence of the real and imaginary parts of the output voltage of the PDP in μ_0H ~ 1 T, and checked the abrupt voltage change at T_c ~ 3 K. Next, we applied two RF pulses corresponding to π/2 and π pulses just before the PDP. The pulse width of each RF pulse is fixed at 5 μs and the voltage of the π/2 pulse is 2/3 of that of the π pulse. The interval is fixed to 40 μs as shown in the inset of Fig. S1. We measured the dependence of the output voltage of the PDP against the power of the two RF pulses in the same condition of the NMR spectrum. To estimate the exact RF-pulse energy absorbed directly by the sample is difficult, and has large ambiguity, we express the power of the NMR RF pulses by the indication of the NMR power amplifier (ML). Figure S1 shows the ML dependence of the output voltage of the PDP, where the voltage was expressed by the Re-Im to make the voltage change clearer. The measurement was done in the SC state at 1.4 K. Although the Re-Im voltage was almost unchanged up to -18 dB, it began to change above -18 dB and, above -11 dB, stayed almost constant with the normal-state value measured at 3.25 K. This indicates that the NMR RF pulses with power above -11 dB immediately destroy the superconductivity of the sample and bring the sample to the normal state above -11 dB. For the present measurements, we applied the NMR RF pulses with the power of -22 dB not to give any effect to the superconductivity.

^{125}Te-NMR spectrum broadening in SC3

Figure S2 shows the T variation of the ^{125}Te-NMR spectrum of Te(1) and Te(2) below 3 K as a function of K - K_{peak}. The ratio between the FWHM of the Te(1) and Te(2) spectra in SC3 is almost unchanged from that in the normal state. Taking into account that the additional linewidth broadening in SC3 is proportional to the applied field, it is considered that the additional linewidth broadening in SC3 arises from the inhomogeneity of the spin susceptibility, not from the static internal field. In general, when the static internal field appears, it depends on the atomic sites, and thus the ratio would be different from that in the normal state.

Figure S3 shows the simulation of the Te(2) NMR spectrum in SC3. To consider the linewidth broadening in SC3 more quantitatively, we have performed the following simulations. At first, as a simple assumption, we consider a two-phase coexistence, which means that a part of the sample remains in the SC2 phase and a part in the SC3 phase. This possibility is easily ruled out from the following comparison of the shape of the spectrum changes. Figure S3A shows a simulation of the simple phase separation. The right-side dotted Lorentz function is the Lorentz function which fits the spectrum measured at 0.7 K. The left side dotted Lorentzian is shifted in an attempt to better explain the 0.13 K data. Such simulations produce flat-top spectra and cannot reproduce experimental spectrum. The experimental spectrum cannot be reproduced by two Lorentz functions. Next, we consider the inhomogeneity in the sample. We assume the inhomogeneity has gaussian distribution. Figures S3B and S3C show a simulation for the 0.13 K spectrum assuming

the gaussian distribution of the 0.7 K spectrum. The simulation in Fig. S3B assumes a Gaussian function with σ=0.02 as the distribution of the center and intensity of the Lorentz function, and in Fig. S3C a Gaussian function with σ=0.07. The Gaussian function with σ=0.02 has a small intensity of about 6 % of the center at the position of the SC2 phase signal, and the distribution of the Knight shift higher than SC2 is negligible. However, the spectrum reproduced by this distribution is sharper than observed. Conversely, the shape of the spectrum is matched in Fig. S3C. In this case, the spectrum is almost reproduced, but its distribution is physically strange because the weight of the Knight shift, which is higher than that of SC2, is quite large. This means that a simple Gaussian distribution cannot explain the broadening of the linewidth.

The phase diagram determined form our NMR measurements are shown in Fig. S4.

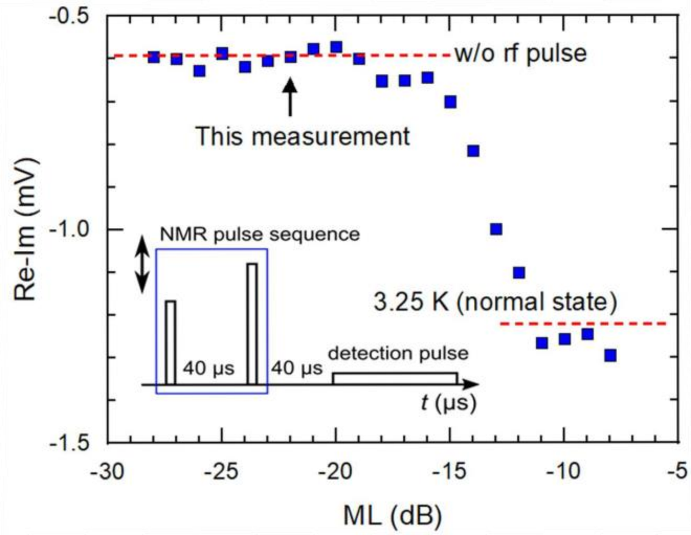

Fig. S1. Heat-up test of the NMR RF pulses.

The dependence of the output voltage of the phase-detection pulses against, where the voltage was expressed by the Re-Im to make the voltage change clearer. The measurement was done in the SC state at 1.4 K. For the present measurements, we applied the NMR RF pulses with the power of -22 dB (shown by arrow) not to give any effect to the superconductivity. The inset shows the schematic of the NMR pulse sequence. To check the sample heating, we change the voltage of two pulses surrounded by the blue box.

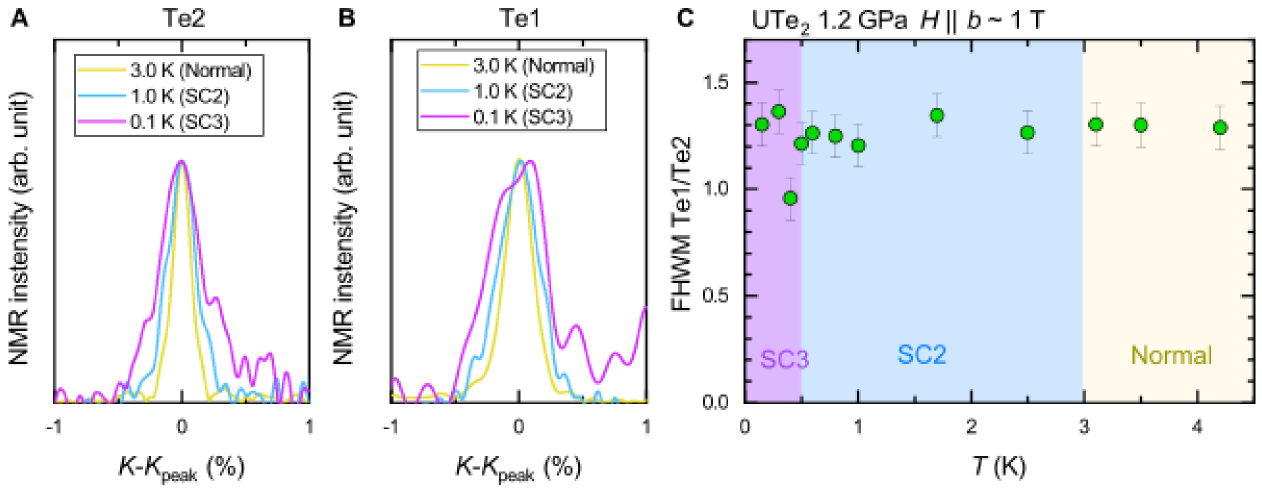

Fig. S2. Evidence for magnetic inhomogeneous superconductivity.

NMR signals at several temperatures are shown in panel (A) and panel (B). (C) The temperature dependence of the ratio of FWHM of two Te sites. FWHM Te1/Te2 is almost temperature-independent, indicating the inhomogeneity of the spin susceptibility.

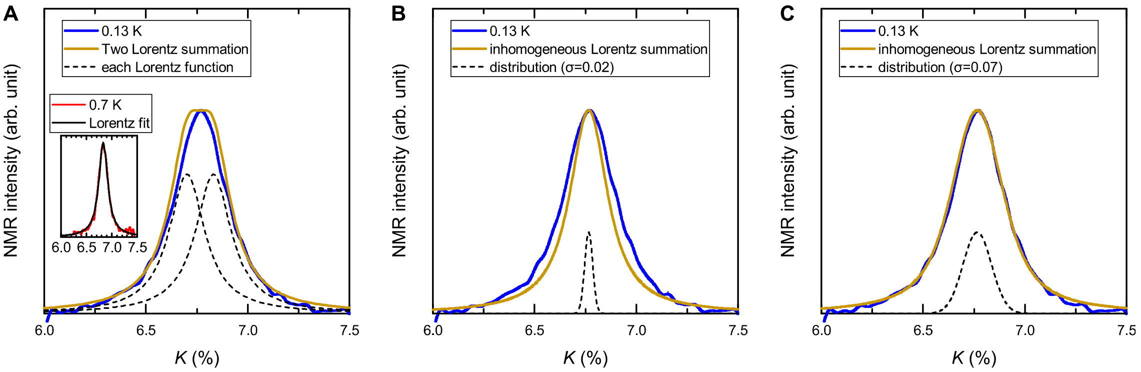

Fig. S3. The simulation of the NMR spectrum in SC3.
(A) The NMR spectrum of the SC3 phase and the spectrum expected when the SC1 and SC2 phases are phase-separated in real space (Two-Lorentz summation). In the model of phase separation, an equal amount of mixing would result in a flat-top, anisotropic spectrum depending on the fraction of either phase, which is different from a symmetrically broad spectrum as obtained in the experiment. The inset is the spectrum just above the SC3 transition used as a model for the Lorentz function. (B)(C) Simulations for the 0.13 K spectrum assuming the normal distribution of the 0.7 K spectrum. (B) Spectrum was calculated with normal distribution with $\sigma = 0.02$. (C) Spectrum was calculated with normal distribution with $\sigma = 0.07$.

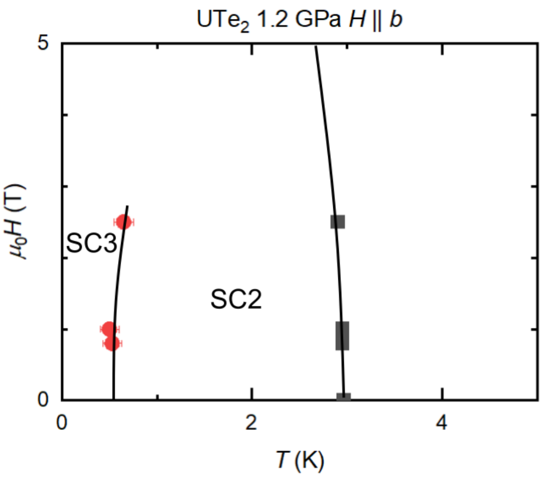

Fig. S4. Phase diagram from NMR measurements.

To determine the phase boundary between the normal and SC2 states, we used the χ_{AC} measurements. To determine the phase boundary between the SC2 and SC3 states, the ΔK shows a clear anomaly as shown in Fig. 3.

REFERENCES AND NOTES

1. A. J. Leggett, A theoretical description of the new phases of liquid ^{3}He. *Rev. Mod. Phys.* **47**, 331–414 (1975).

2. J. C. Wheatly, Experimental properties of superfluid ^{3}He. *Rev. Mod. Phys.* **47**, 415–470 (1975).

3. H. Kojima, H. Ishimoto, Spin polarized superfluid ^{3}He A_1. *J. Phys. Soc. Jpn.* **77**, 111001 (2008).

4. V. V. Dmitriev, M. S. Kutuzov, A. A. Soldatov, A. N. Yudin, Superfluid β phase of ^{3}He. *Phys. Rev. Lett.* **127**, 265301 (2021).

5. S. Murakawa, Y. Wada, Y. Tamura, M. Wasai, M. Saitoh, Y. Aoki, R. Nomura, Y. Okuda, Y. Nagato, M. Yamamoto, S. Higashitani, K. Nagai, Surface Majorana cone of the superfluid ^{3}He B phase. *J. Phys. Soc. Jpn.* **80**, 013602 (2011).

6. R. Joynt, L. Taillefer, The superconducting phases of UPt_3. *Rev. Mod. Phys.* **74**, 235–294 (2002).

7. G. R. Stewart, UBe_{13} and $U_{1-x}Th_xBe_{13}$: Unconventional superconductors. *J. Low Temp. Phys.* **195**, 1–25 (2019).

8. S. Khim, J. F. Landaeta, N. Bannor, M. Brando, P. M. R. Brydon, R. Cardoso-Gil, U. Stockert, A. P. Mackenzie, D. F. Agterberg, C. Geibel, E. Hassinger, Field-induced transition within the superconducting state of $CeRh_2As_2$. *Science* **373**, 1012–1016 (2021).

9. H. Tou, Y. Kitaoka, K. Ishida, K. Asayama, N. Kimura, Y. Onuki, E. Yamamoto, Y. Haga, K. Maezawa, Nonunitary spin-triplet superconductivity in UPt_3: Evidence from ^{195}Pt Knight shift study. *Phys. Rev. Lett.* **80**, 3129–3132 (1998).

10. S. Ran, C. Eckberg, Q.-P. Ding, Y. Furukawa, T. Metz, S. R. Saha, I.-L. Liu, M. Zic, H. Kim, J. Paglione, N. P. Butch, Nearly ferromagnetic spin-triplet superconductivity. *Science* **365**, 684–687 (2019).

11. D. Braithwait, M. Vališka, G. Knebel, G. Lapertot, J.-P. Brison, A. Pourret, M. E. Zhitomirsky, J. Flouquet, F. Honda, D. Aoki, Multiple superconducting phases in a nearly ferromagnetic system. *Commun. Phys.* **2**, 147 (2019).

12. S. Ran, H. Kim, I.-L. Liu, S. R. Saha, I. Hayes, T. Metz, Y. S. Eo, J. Paglione, N. P. Butch, Enhancement and reentrance of spin triplet superconductivity in UTe_2 under pressure. *Phys. Rev. B* **101**, 140503 (2020).

13. S. M. Thomas, F. B. Santos, M. H. Christensen, T. Asaba, F. Ronning, J. D. Thompson, E. D. Bauer, R. M. Fernandes, G. Fabbris, P. F. S. Rosa, Evidence for a pressure-induced antiferromagnetic quantum critical point in intermediate-valence UTe_2. *Sci. Adv.* **6**, eabc8709 (2020).

14. G. Knebel, W. Knafo, A. Pourret, Q. Niu, M. Vališka, D. Braithwaite, G. Lapertot, J.-P. Brison, S. Mishra, I. Sheikin, G. Seyfarth, D. Aoki, J. Flouquet, Field-reentrant superconductivity close to a metamagnetic transition in the heavy-fermion superconductor UTe_2. *J. Phys. Soc. Jpn.* **88**, 063707 (2019).

15. A. Rosuel, C. Marcenat, G. Knebel, T. Klein, A. Pourret, N. Marquardt, Q. Niu, S. Rousseau, A. Demuer, G. Seyfarth, G. Lapertot, D. Aoki, D. Braithwaite, J. Flouquet, J. P. Brison, Field-induced tuning of the pairing state in a superconductor. *Phys. Rev. X* **13**, 011022 (2023).

16. K. Kinjo, H. Fujibayashi, S. Kitagawa, K. Ishida, Y. Tokunaga, H. Sakai, S. Kambe, A. Nakamura, Y. Shimizu, Y. Homma, D. X. Li, F. Honda, D. Aoki, K. Hiraki, M. Kimata, T. Sasaki, Change of superconducting character in UTe_2 induced by magnetic field. *Phys. Rev. B* **107**, L060502 (2023).

17. G. Nakamine, S. Kitagawa, K. Ishida, Y. Tokunaga, H. Sakai, S. Kambe, A. Nakamura, Y. Shimizu, Y. Homma, D. Li, F. Honda, D. Aoki, Superconducting properties of heavy fermion UTe_2 revealed by ^{125}Te-nuclear magnetic resonance. *J. Phys. Soc. Jpn.* **88**, 113703 (2019).

18. G. Nakamine, K. Kinjo, S. Kitagawa, K. Ishida, Y. Tokunaga, H. Sakai, S. Kambe, A. Nakamura, Y. Shimizu, Y. Homma, D. Li, F. Honda, D. Aoki, Anisotropic response of spin susceptibility in the superconducting state of UTe_2 probed with ^{125}Te–NMR measurement. *Phys. Rev. B* **103**, L100503 (2021).

19. G. Nakamine, K. Kinjo, S. Kitagawa, K. Ishida, Y. Tokunaga, H. Sakai, S. Kambe, A. Nakamura, Y. Shimizu, Y. Homma, D. Li, F. Honda, D. Aoki, Inhomogeneous superconducting state probed by ^{125}Te NMR on UTe_2. *J. Phys. Soc. Jpn.* **90**, 064709 (2021).

20. H. Fujibayashi, G. Nakamine, K. Kinjo, S. Kitagawa, K. Ishida, Y. Tokunaga, H. Sakai, S. Kambe, A. Nakamura, Y. Shimizu, Y. Homma, D. Li, F. Honda, D. Aoki, Superconducting order parameter in UTe_2 determined by Knight shift measurement. *J. Phys. Soc. Jpn.* **91**, 043705 (2022).

21. Y. Tokunaga, H. Sakai, S. Kambe, T. Hattori, N. Higa, G. Nakamine, S. Kitagawa, K. Ishida, A. Nakamura, Y. Shimizu, Y. Homma, D. Li, F. Honda, D. Aoki, ^{125}Te-NMR study on a single crystal of heavy fermion superconductor UTe_2. *J. Phys. Soc. Jpn.* **88**, 073701 (2019).

22. C. Duan, K. Sasmal, M. B. Maple, A. Podlesnyak, J.-X. Zhu, Q. Si, P. Dai, Incommensurate spin fluctuations in the spin-triplet superconductor Candidate UTe_2. *Phys. Rev. Lett.* **125**, 237003 (2020).

23. W. Knafo, G. Knebel, P. Steffens, K. Kaneko, A. Rosuel, J.-P. Brison, J. Flouquet, D. Aoki, G. Lapertot, S. Raymond, Low-dimensional antiferromagnetic fluctuations in the heavy-fermion paramagnetic ladder compound UTe_2. *Phys. Rev. B* **104**, L100409 (2021).

24. D. Li, A. Nakamura, F. Honda, Y. J. Sato, Y. Homma, Y. Shimizu, J. Ishizuka, Y. Yanase, G. Knebel, J. Flouquet, D. Aoki, Magnetic properties under pressure in novel spin-triplet superconductor UTe_2. *J. Phys. Soc. Jpn.* **90**, 073703 (2021).

25. K. Kinjo, H. Fujibayashi, G. Nakamine, S. Kitagawa, K. Ishida, Y. Tokunaga, H. Sakai, S. Kambe, A. Nakamura, Y. Shimizu, Y. Homma, D. Li, F. Honda, D. Aoki, Drastic change in magnetic anisotropy of UTe_2 under pressure revealed by ^{125}Te-NMR. *Phys. Rev. B* **105**, L140502 (2022).

26. G. Knebel, M. Kimata, M. Vališka, F. Honda, D. Li, D. Braithwaite, G. Lapertot, W. Knafo, A. Pourret, Y. J. Sato, Y. Shimizu, T. Kihara, J.-P. Brison, J. Flouquet, D. Aoki, Anisotropy of the upper critical field in the heavy-fermion superconductor UTe_2 under pressure. *J. Phys. Soc. Jpn.* **89**, 053707 (2020).

27. R. F. Hoyt, H. N. Scholz, D. O. Edwards, Superfluid ^{3}He-B: The dependence of the susceptibility and energy gap on magnetic field. *Physica B+C* **107**, 287–288 (1981).

28. Supporting information are available as Supplementary Materials.

29. N. R. Werthamer, E. Helfand, P. C. Hohenberg, Temperature and purity dependence of the superconducting critical field, H_{c2}. III. Electron spin and spin-orbit effects. *Phys. Rev.* **147**, 295 (1966).

30. R. H. Heffner, J. L. Smith, J. O. Willis, P. Birrer, C. Baines, F. N. Gygax, B. Hitti, E. Lippelt, H. R. Ott, A. Schenck, E. A. Knetsch, J. A. Mydosh, D. E. MacLaughlin, New phase diagram for (U,Th)Be_{13}: A muon-spin-resonance and H_{C1} study. *Phys. Rev. Lett.* **65**, 2816 (1990).

31. S. K. Ghosh, M. Smidman, T. Shang, J. F. Annett, A. D. Hiller, J. Quintanilla, H. Yuan, Recent progress on superconductors with time-reversal symmetry breaking. *J. Phys. Condens. Matter* **33**, 033001 (2021).

32. M. Sigrist, T. M. Rice, Phenomenological theory of the superconductivity phase diagram of U1−xThx. *Phys. Rev. B Condens Matter* **39**, 2200–2216 (1989).

33. S. Imajo, Y. Kohama, A. Miyake, C. Dong, M. Tokunaga, J. Flouquet, K. Kindo, D. Aoki, Thermodynamic investigation of metamagnetism in pulsed high magnetic fields on heavy fermion superconductor UTe_2. *J. Phys. Soc. Jpn.* **88**, 083705 (2019).

34. A. Miyake, Y. Shimizu, Y. J. Sato, D. Li, A. Nakamura, Y. Homma, F. Honda, J. Flouquet, M. Tokunaga, D. Aoki, Metamagnetic transition in heavy fermion superconductor UTe_2. *J. Phys. Soc. Jpn.* **88**, 063706 (2019).

35. D. Aoki, A. Nakamura, F. Honda, D. Li, Y. Homma, Y. Shimizu, Y. J. Sato, G. Knebel, J.-P. Brison, A. Pourret, D. Braithwaite, G. Lapertot, Q. Niu, M. Vališka, H. Harima, J. Flouquet, Unconventional superconductivity in heavy fermion UTe_2. *J. Phys. Soc. Jpn.* **88**, 043702 (2019).

36. N. Fujiwara, N. Môri, Y. Uwatoko, T. Matsumoto, N. Motoyama, S. Uchida, Superconductivity of the $Sr_2Ca_{12}Cu_{24}O_{41}$ spin-ladder system: Are the superconducting pairing and the spin-gap formation of the same origin? *Phys. Rev. Lett.* **90**, 137001 (2003).

37. B. Bireckoven, J. Witting, A diamond anvil cell for the investigation of superconductivity under pressures of up to 50 GPa: Pb as a low temperature manometer. *J. Phys. E Sci. Instrum.* **21**, 841–848 (1988).

38. K. Kitagawa, H. Gotou, T. Yagi, A. Yamada, T. Matsumoto, Y. Uwatoko, M. Takigawa, Space efficient opposed-anvil high-pressure cell and its application to optical and NMR measurements up to 9 GPa. *J. Phys. Soc. Jpn.* **79**, 024001 (2010).

39. K. Momma, F. Izumi, *VESTA 3* for three-dimensional visualization of crystal, volumetric and morphology data. *J. Appl. Crystallogr.* **44**, 1272–1276 (2011).

40. A. Pustogow, Y. Luo, A. Chronister, Y.-S. Su, D. A. Sokolov, F. Jerzembeck, A. P. Mackenzie, C. W. Hicks, N. Kikugawa, S. Raghu, E. D. Bauer, S. E. Brown, Constraints on the superconducting order parameter in Sr_2RuO_4 from oxygen-17 nuclear magnetic resonance. *Nature* **574**, 72–75 (2019).

41. K. Ishida, M. Manago, K. Kinjo, Y. Maeno, Reduction of the ^{17}O Knight shift in the superconducting state and the heat-up effect by NMR pulses on Sr_2RuO_4. *J. Phys. Soc. Jpn.* **89**, 034712 (2020).

42. K. Kinjo, M. Manago, S. Kitagawa, Z. Q. Mao, S. Yonezawa, Y. Maeno, K. Ishida, Superconducting spin smecticity evidencing the Fulde-Ferrell-Larkin-Ovchinnikov state in Sr_2RuO_4, *Science* **376**, 397–400 (2022).
